# Transcribing in the digital age: qualitative research practice utilizing intelligent speech recognition technology

**DOI:** 10.1093/eurjcn/zvae013

**Published:** 2024-02-05

**Authors:** Helen Eftekhari

**Affiliations:** Department of Health Sciences, University of Warwick, UK; Department of Cardiology, Institute for Cardio-Metabolic Medicine, University Hospitals Coventry and Warwickshire NHS Trust, UK

**Keywords:** Digital age, Qualitative methods, Simultaneous speech recognition technology, Transcribing

## Abstract

The digital revolution provides many opportunities for researchers to develop and evolve data collection methods. A key process in qualitative research data collection is the transcription of interviews, focus groups or fieldwork notes. Transcription is the process of converting audio, video or notes into accessible written format for qualitative data analysis. Transcribing can be time intensive, costly and laborious with decisions and methods of transcribing requiring transparency. The development of intelligent speech recognition technology can change how qualitative data is transcribed. This methods paper describes audio data transcribing, current challenges, opportunities and implications in using intelligent speech recognition technology for transcribing. An application of this methodology is presented.

## Graphical Abstract

**Graphical Abstract:**
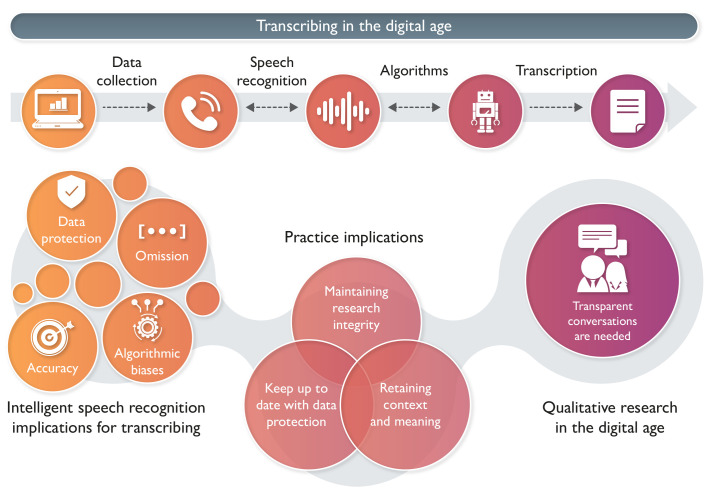

**Learning objectives**

- Identify issues related to transcribing textual qualitative data.
- Identify key benefits and challenges of using intelligent speech recognition technology for transcription.
- Explore future implications associated with the use of intelligent speech recognition technology.

## Introduction

With the digital revolution, we have seen rapid development in the use of digital technology in the fields of health care and research methods. A rapid accelerator for digital technology applications came in response to social restrictions during the COVID-19 pandemic.^1,2^ Qualitative research applications of digital technology range from online data collection such as interviewing,^2^ to the use of intelligent speech recognition technology.^3^ Intelligent speech recognition technology refers to ‘*the use of machine learning methods to process human voice signals to obtain information and realize human-machine communication*’.^1^ The growing use of these technologies can be seen in many areas, such as electronic chat-bots or electronic medical documentation and reporting systems.^1,4^ When transcribing algorithm-based systems make decisions on who is speaking, how to enhance speech quality or eliminate background noise, and what grammar to use. Transcribing with intelligent speech recognition technology is an evolving science.

This paper explores the challenges and benefits of using intelligent speech recognition to transcribe interviews. We also present an application of this methodology from a study on supportive self-management in Postural Orthostatic Tachycardia Syndrome (also known as POTS).

## Overview of transcribing methodology

Transcribing and transcribing decisions are a key aspect of robust qualitative research methods. The overall aim being to have data accurately transcribed to the best quality, to facilitate robust data analysis and development of concepts and themes supported by thick, rich, contextual qualitative data. Transcribing converts raw data from audio, video or fieldnotes into an accessible written format suitable for data management, analysis and an auditable trail.^5^ Technology for recording and transcribing has evolved from analogue equipment to digital recorders, foot controlled transcription machines to transcribing software and in the modern era to digital synchronous recording and transcribing.^6^ Each research project requires key strategic transcription decisions (see *Table 1*) about the necessity of transcription, how much to transcribe, who will transcribe, and what transcription medium to use.^3,7^ How much raw data to transcribe can be guided by differing analytical approaches. Conversational analysis, for instance, requires a high level of detailed transcription, and other analytical approaches such as latent conversational analysis, may require translation of non-verbal communication like facial expressions.^8^

**Table 1:** Methods in conventional transcribing practices (compiled from^3,6,9–11^)

What	Example	Consideration	
Level of transcription required	Transcription omission (i.e. no transcription)	Does direct coding of raw data have an impact on quality of analysis Is there a need for quick turnaround of findings	
	Verbatim transcription of data	Provides a word for word account of your data	
	Conversation analysis	Requires a high level of detail	
	Whether to include non-verbal elements	Dependent on aims of research & chosen analytical strategy	
Nomenclature indicating participants in data	I (for interviewer) P (for participant and a number)	Does the nomenclature maintain pseudo-anonymity Use at start of each new utterance indicating roles	
	Pseudonym name	Does this ascribe a role, gender or identity or power relation HCP = health care practitioner P = person with a condition	
When to start new line	I: ‘*How are you*’ P: ‘*I’m well*’	When each new speaker starts	
	Significant pauses	Should there be a new line denoting significant silence	
	Line numbers	For easy referencing	
Wide margins		Space for coding or notes	
Punctuation, Grammar, and homonyms	*‘Eat your dinner*’ vs. ‘*Eat, you’re dinner!*’ ‘*your*’ vs. ‘*you’re*’, ‘*there*’ vs. ‘*their*’ vs. ‘*they’re*’	Consider speaker meaning and context to inform	
Hyphen	Wo- or Becau-	Indicates a word interrupted/cut off	
Use of brackets	[ ]	Overlapping talking or emotion	
Use of parentheses	(a guesstimated word)	Transcriber estimating at unclear word or using generic term preserving pseudonymity	
	(…..)	Unclear words or omission of identifier	
	(?)	Unclear word	
All of a word capitalized	WORD	Words spoken more loudly or sounds	
Who transcribes		**Advantage**	**Disadvantage**
	Primary researcher	Detailed contextual knowledge for informed transcribing decisions Data immersive	Time consuming Human resource intensive
	Junior researcher/secretary	Can access contextual knowledge and time efficient	Detailed contextual knowledge absent
	Outsourced	Time efficient Frees up mechanistic typing aspects	Costly Risk of biases and errors requiring careful checking (minimized by verbatim transcription) No contextual knowledge
	Digital Tools	Time efficient, quick access, frees up mechanistic typing aspects Reduces cost	Emerging technology prone to accuracy & bias errors requiring detailed checking
Conventions for presenting transcribed extracts			
Square enclosed brackets	[data]	Data added by author	
	[…]	Data omitted by author	
How much to extract	Where to begin or end	Correct account of context and data evidence	

### Transcription as translational

Verbatim transcription is a translational and dynamic process involving more than mechanistic thinking. General transcribing convention exists (see *Table 1*) and involves reproducing reliably precise words used by participant/s, including slang words, stutters, hesitations, and interruptions.^9^ However, transcribing is more than words on a page. Words express meaning relying on punctuation, grammar, and word organization.^6,9^ Slight variations in grammar, spelling or punctuation changes meaning, for instance ‘*Eat your dinner*’ vs. ‘*Eat. You’re dinner!*’.^11^ Inaccurate inferences can occur as even verbatim transcription may not represent exactly what a person meant to say. Transcribing involves an understanding of context.^6^ Researchers interpret words drawing upon their own knowledge and perspectives to inform transcribing decisions.^7,12^ Hence, researchers should always check the transcript against the audio recordings. Everyday conversation is seldom grammatical or conducted in complete sentences and transcriptions should reproduce the conversation as it happened. Transcribing and transcription checking require active thinking and decision making, informed by researcher’s chosen analytical method. With the emergence over the past decade of algorithmic intelligent speech recognition tools, this raises questions around transcribing raw data and the impact on the qualitative research process.

## Overview of new methodological approaches in transcribing

Innovation in digital technology is now changing the possibilities of how researchers choose to record and transcribe. Two key developments are (1) the use of algorithmic speech-recognition technology for synchronous transcription and (2) data management systems offering possibilities for transcription omission.

### Algorithmic speech-recognition systems

Modern day intelligent speech-recognition systems use algorithms to inform transcribing decisions. Algorithmic systems, are evolving, particularly in medical care.^13^ Intelligent speech recognition systems are being implemented, continually developed, and are increasing in their sophistication. These systems open up possibilities for research applications. Speech recognition systems improve communication with people with speech impediments, can detect emotional states and be used to record medical emergency scenarios.^1^ Data can be gathered from emergency scenarios with accurate timings and voice recordings, people with ranges of speech impediments (such as those with aphasia after stroke) can be included in interviews or focus groups and emotional analysis techniques adjusted. The technology is quickly developing with a crucial need for researchers to explore the implications for research.

### Transcription omission

Current qualitative data analysis systems offer the possibility of omitting transcribing by direct coding from files. These systems facilitate direct coding from raw data sources images such as videos, speeches or texts,^3^ supporting researchers with managing large and varied data sets for their analysis. Advantages of direct coding audio files could include cost-savings, facilitating management of visual data like photographs or charts or remaining close to the data through the constant hearing of the participant’s voice.^14^ Direct coding and transcript omission are currently not widely used. It is proposed that direct coding could hinder the initial stage in the qualitative process of data immersion, that is familiarizing and gaining a deep understanding of what is being said.^3,14^ Halcombe and Davidson^5^ propose iterative analysis and direct coding of good quality audio recordings, concurrent field notes and a reflective journal can provide and increase representation of values and beliefs of participants.^5^ As technology moves forward, the question around transcription omission (i.e. skipping the time consuming and laborious process of converting raw data into a written format) will become progressively pertinent for qualitative researchers.

### Advantages of new methodological approaches

There are advantages of transcribing using these new methodological approaches. Using intelligent speech recognition technology can reduce mechanistic typing, provide immediate access to the transcript and reduce costs.^3^ Costs for professional external transcribing can be expensive and is often paid for by competitive research funding. Many novice qualitative researchers (such as PhD students) however may not have access to funds and transcribe themselves. Transcribing is a time consuming laborious task with an estimated 3–10 h to transcribe 1 h of raw data.^1,3,5^ Advantages of transcription omission can be rapid data turnaround to inform policy, practice and take account of current public opinion.^15^ For instance, during COVID-19 a pressing need existed to rapidly capture a deep understanding of life during the pandemic to inform policies and practices.^16^

### Challenges

There are key challenges in using algorithmic speech recognition systems from data protection, algorithmic biases, and accuracy.

#### Data protection

Protecting our individual data and right to confidentiality is a fundamental debate of the digital era. Researchers need to work within data protection systems for data security and transcription storage.^17^ Data protection systems are required to comply with international and national regulations which enshrine data protection legislation and rights.^18,19^ Key personal data principles includes agreements for consenting and confidentiality, being specific about what data is collected, processing data lawfully and transparently, and transferring data securely through agreed methods.^17,19^ Many countries have national data regulation guidelines for developers and adopters of digital technology, including for health care or social settings.^18^ Based upon these regulations, local institutions then develop their digital protocols and data protection systems.

Intelligent speech recognition generally requires utilizing a tech-company’s third party platform. For example, Microsoft Teams or Google Cloud speech to text. Researchers using third party platforms need to maintain data protection awareness, and meet governance and legislative requirements around these principles. Even with the digital regulations^18,19^ controversy remains over flaws in legislation and the ability of companies, particularly large technology ones, to circumnavigate data protection.^20^ Although research data is generally stored with organizations that have high levels of data security, these systems are increasingly vulnerable to breaches in security, cybercrime, and data hacking.^21^ As with all research, and especially those using intelligent speech recognition systems, it is necessary to guard against breaches in participants’ confidentiality during data recording, retrieval, and storage of transcriptions, be compliant with data protection policies, and report suspected breaches.

#### Algorithmic biases

Intelligent speech recognition tools make decisions, including which words to use, spelling, punctuation, and grammar. Artificial intelligence and algorithm programming are not immune to bias as decision-making is based completely on the data humans input which is prone to errors.^22^ When utilizing artificial intelligent tools in qualitative research, researchers need to be alert to biases within algorithmic-based systems. Researchers need to check transcription accuracy against audio or video recordings and ensure a robust data analysis by maintaining and checking context and participant’s meaning. As systems develop, it is incumbent on researchers to be transparent on the issues and implications current technology presents for maintaining research integrity.

#### Algorithmic speech-recognition technology’s accuracy

A more practical issue in modern transcription tools is lack of accuracy. Error checking, formatting, and corrections could increase transcription turnaround.^1,3^ To minimize accuracy error, it is important to have good quality audio recording, by using headsets to minimize echoes, trying to minimize overlapping speech, and having a quite environment to minimize background noise.^3^

## Example application

The study of the experiences of people with POTS and health care practitioners utilized intelligent speech technology for transcribing. This is presented as an example illustrating practical benefits and challenges of using algorithmic technology for transcribing interviews in a qualitative study.

### Using online interviewing medium transcription software for simultaneous transcribing

In response to the COVID-19 pandemic and in line with many qualitative projects,^2^ interviewing was conducted online between October 2021 and September 2022. The study was a funded research project receiving University sponsorship and United Kingdom Health Research Authority ethical approval (HRA IRAS number 281284). The university approved medium for online interviewing at the time was Microsoft Teams (Microsoft Corporation, Redmond, WA, USA). A total of 44 people were interviewed. Interviews were topic guided and in-depth with interview duration ranging from 40 to 131 min and an average length of 65 min. Within the Teams platform is an option to record and transcribe simultaneously, with almost instantaneous access to the transcript following an interview. Artificial speech-recognition technology transcribed 41 of the in-depth interviews, and the remaining three interviews were transcribing by an external company (one person preferred a telephone interview and two interviews had technical issues accessing the platform). Using this speech-recognition technology for transcribing provided both benefits and challenges.

The ‘how to approach’ and step-by-step guide is presented in *Graphical Abstract* which has embedded brief videos to support other researchers who are considering this method. Transcription files were immediately stored on the platform after interviews; then they were downloaded, password-protected, and stored in a secure university cloud drive and deleted from the platform (*Videos 1–4*).

### Benefits of simultaneous transcribing during the interviews

Using intelligent speech-recognition software for transcribing provided tangible benefits in the experience of POTS study. Firstly, the typed transcription was almost immediately available and having the timely availability of the transcript facilitated a more engaging data immersion. Transcripts required checking against the audio recordings meaning and the researcher had to continually think about the meaning and context of what was said and question if the technology generated transcript accurately reflected these. In contrast, outsourced transcriptions (*n* = 3) had a time lag in turnaround (in one case 10 days), and although a quick turnaround could be requested, this would have incurred additional costs. All transcripts required checking and cleaning. In the case of the simultaneous generated transcriptions, transcript checking could begin almost immediately following interviews. However, these transcripts required the most amount of accuracy checking, between 1.5 and 3.5 h depending on accuracy and interview length. Accuracy checking for context and meaning with the simultaneous transcriptions proved to be more data immersive, reflective, and analytical in the early stage of thematic identification.

A crucial benefit to this approach in this case study was cost savings. An estimated £3772 was saved from allocated transcribing costs. Funding could be re-allocated to unforeseen expenditure in the research, saving funders additional expenses.

### Challenges in the transcripts

The two main challenges arising from the 41 algorithmic speech recognition transcriptions were (1) the accuracy of dialogue and (2) the need for document formatting. Accuracy of the algorithmic speech recognition transcriptions was generally poorer and took longer than the externally outsourced transcriptions. Although, outsourcing transcribers also took occasional decisions to omit data they thought irrelevant, necessitating careful checking with the audio recording and inserting back the data to inform the context of discussion. Even with paid external professional transcribing, researchers still need to check and clean documents and ensure all identifying information is removed.

#### Dialogue accuracy

Accurate transcription of interviewee dialogue varied depending on interviewees colloquial accents and/or, dialects, speaking tempos, and pronunciation inflections. Colloquial accents and dialect presented an interesting challenge for the speech technology. The technology generally transcribed accurately southern (*n* = 16) and midlands (*n* = 3) English dialects, however northern English dialects (*n* = 23), had more transcription inaccuracies. Tempo and pronunciation impacted accuracy. In some dialects, Irish (*n* = 1) and Scottish (*n* = 1) the interviewees pronounced slowly and clearly resulting in better transcription accuracy. Some interviewees spoke at great speed or without pausing or variations in pronunciation. The speech recognition technology transcribed speed talking fairly accurately, however transcriptions tended to be more inaccurate combined with either northern accents and, or pronunciation variations. For instance, an interviewee with fast quiet pronunciations often spoke of ‘*clinicians*’; frequently mis-transcribed as either ‘*collide*’, ‘*collusions*’, ‘*confide*’ or ‘*commission*’. Finally, an interesting example of bias in algorithms arose transcribing the word ‘*target*’, always transcribed by the intelligent speech recognition technology with a capital ‘T’. The context of target in the interviews commonly referenced health targets, however the capital ‘T’ in the transcription denotes a proper noun, an American shopping chain named ‘*Target*!’

#### Grammatical accuracy

Transcription grammar could be a challenge principally related to the use of punctuation, speech fillers (*um*, *er*, *ah*) and homophones. Secondary issues included word repetitions and or transposing words. In the speech recognition transcriptions, frequent punctuation errors occurred resulting from either sentences being clipped short by inappropriate use of full stops during conversational pauses or resulting from a high sensitivity to speech fillers. For instance, the interviewer uttering ‘*uh-hmm*’ to signal listening and encouraging an interviewee to continue, generally in transcription resulted in a new paragraph of one word ‘*uh-hmm*’. Careful checking was required when listening to audio recordings, ensuring context and meaning were accurate and maintained.

Homophones, words that sound alike and are spelled differently, presented particular conundrums for algorithmic decision-making. First was the use of unfamiliar words; for example, the health condition ‘*PoTS*’, was always transcribed as ‘*parts*’, the drug ‘*ivabradine*’ always ‘*Aberdeen*’, a Scottish city, or ‘*prostate*’ transcribed as ‘*prostitute*’. Second were the more subtle homophone inaccuracies requiring algorithmic decisions around context and meaning. Subtle homophone inaccuracies included the use of ‘*there*’, ‘*their*’ or ‘*they*’*re*’; ‘*your*’ or ‘*you*’*re*’ and ‘*role*’ or ‘*roll*’. Nonetheless, intelligent speech technology accuracy could be high, words transcribed and spelled correctly included ‘*autonomic nervous system*’, ‘*dysautonomia*’, ‘*Munchausen*’*s*’ or ‘*COVID*’ and homophones in transcriptions often reflected the correct context.

#### Transcription formatting

A key challenge was the format of the autogenerated transcription. The transcription document could be downloaded as either a word document or in an automatic cleaner format, a video tracked text (VTT) file. Following the initial interviews, the word document was download, an often substantially lengthy document. Document length resulted from frequent unnecessary paragraphs due to a combination of high sensitivity to speech fillers and the use of a timestamp at every paragraph. Revising the document to a manageable length was achieved during accuracy checking. Liaising with the local information technology department provided a partial solution to this in the latter interviews by downloading the VTT file. Although this removed the timestamp, and significantly shortened the document length, the VTT document was one thick block of text for the whole interview, again requiring formatting during accuracy checking. Overall, the VTT file provided an easier format to work with. In general, all transcripts needed cleaning up, formatting, checking for meaning, spelling, grammar, and removal of interviewee identifiers however this was generally longer in the algorithmic speech recognition produced transcripts.

**Figure 1:**
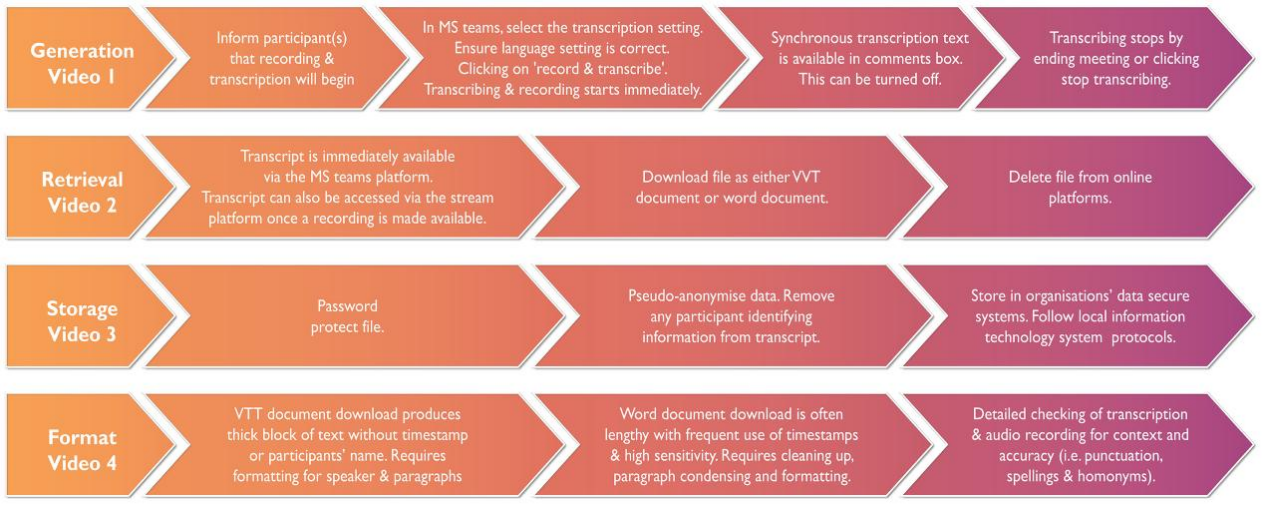
Transcribing issues with intelligent speech recognition technology.

In summary both benefits and challenges exist in using current speech recognition technologies. Artificial speech recognition technology does raise the possibility of a balance between quick transcription turnaround for early career researchers, minimizing the tedious task of typing, whilst focusing on initial data immersion and maintaining context. *Figure 1* summarizes key considerations when using artificial speech recognition technology.

**Figure 2:**
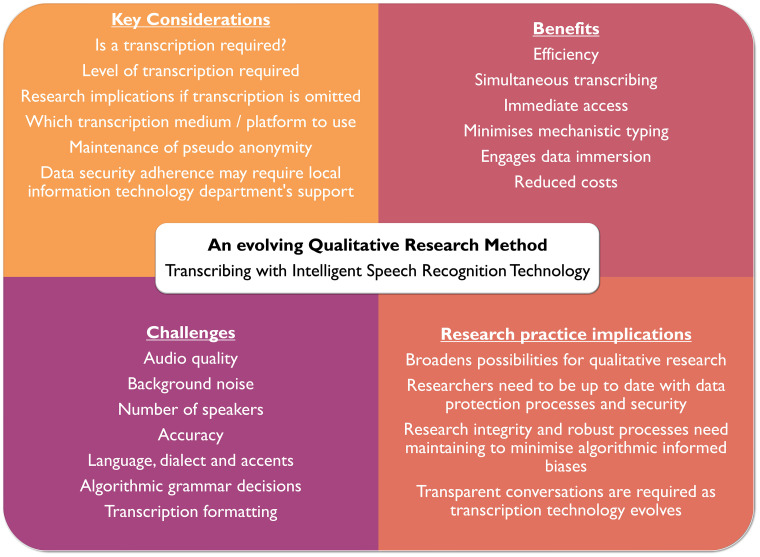
Key considerations for transcribing in the digital age.

*Figure 2* visualizes transcribing with intelligent speech recognition technology issues, from key considerations, benefits, challenges, and research practice implications.

## Conclusion

Using artificial intelligence systems for qualitative research transcriptions broadens possibilities. Advancing technology is changing the landscape for transcription, from more inclusive research to rapid turnaround and research reporting. Qualitative researchers may need to re-think their methods and tools of analysis, particularly as artificial speech recognition systems are continually advancing. It is incumbent upon researchers to be engaged in discussions and be transparent around evolving transcription methods and their research implications.

## Data Availability

No new data were generated or analysed in support of this research.
